# A new mutation in the SERPING1 gene in a Brazilian family with hereditary angioedema

**DOI:** 10.1186/1939-4551-8-S1-A116

**Published:** 2015-04-08

**Authors:** Jane Da Silva, Jéssica Goedert Pereira, Edelton Flávio Morato, Maria Madalena Luz, Nathália Cagini, Camila Lopes Veronez, João Bosco Pesquero

**Affiliations:** 1 University of Southern Santa Catarina - Unisul, Brazil; 2 Hospital Universitário Polydoro Ernani De São Thiago - HU/Ufsc, Brazil; 3 Universidade Federal De Santa Catarina – Ufsc, Brazil; 4 Centro De Ciências Biológicas - CCB/Ufsc, Brazil; 5 Universidade Federal De São Paulo - Escola Paulista De Medicina, Brazil

## Background

Hereditary angioedema (HAE) types I and II (due to quantitative and qualitative C1-INH deficiency, respectively) is a rare autosomal dominant condition in which more than 300 different mutations in the entire C1-INH gene (SERPING1) have been described. The objective of this study is to identify and characterize the mutation in the SERPING1 gene in a family of HAE outpatients from the allergy service of the University Hospital at Universidade Federal de Santa Catarina.

## Methods

DNA was extracted from peripheral blood of 12 symptomatic members of the same family. All the eight exons, the flanking regions and splicing sites of the SERPING 1 gene were analyzed by Sanger sequencing. Analysis was performed by capillary electrophoresis and the electropherograms produced were aligned against the reference sequence of the SERPING1 gene in the GenBank (Accession Number NG_009625.1).

## Results

Subjects age ranged from 4 to 58 years (33 + 15 years), composed by 11 females (n=91,7%) and 1 male (n=8,3%). DNA sequencing revealed a new mutation in the exon 7 of the SERPING1 gene, a deletion of one single base in heterozygosis (c.1104delA) leading to the frameshift alteration p.D69*fs*X96. This mutation was found in seven of the 12 patients (all females), all of them presenting clinical symptoms and low C1-INH plasma levels. The other five family members who reported themselves as symptomatic did not show altered levels of C4, C1q, or C1INH, and gene mutation was not found in these subjects.

## Conclusions

This study allowed the establishment of the molecular basis of a type I HAE in a Brazilian family. The finding of a SERPING1 gene mutation will allow better diagnosis and genetic counseling in the other members of the family.

